# Emotional intelligence in medical students at the University of Ghana Medical School, Accra, Ghana

**DOI:** 10.4314/gmj.v55i1.8

**Published:** 2021-03

**Authors:** Henry J Lawson, Makafui Yigah, Phaedra Yamson

**Affiliations:** 1 Family Medicine Unit, Dept of Community Health, University of Ghana; 2 Ghana College of Physicians and Surgeons, Accra, Ghana; 3 Department of Surgery, Cape Coast Teaching Hospital, Cape Coast, Ghana; 4 Department of Community Health, University of Ghana, Accra, Ghana

**Keywords:** Emotional Intelligence, Medical Student, Accra, Ghana, EQ

## Abstract

Emotional Intelligence is a form of interpersonal intelligence. There's evidence that high emotional intelligence (EQ) of a medical student is associated with better coping with the tedious medical training. We evaluated EQ in medical students in their clinical years. This cross-sectional study was conducted in the University of Ghana Medical School (UGMS), Accra. In all, 111 students completed the questionnaires. Their average age was 24±1.5 years with marginal male preponderance. There were 37, 31 and 43 students in the 1st, 2nd and 3rd clinical years respectively. For global scores, 16 (14.1%) had good EQ (>120) with a mean total score of 105.49. The mean EQ for males was 104.2±17 and 106.2±12 for females, however this difference was not statistically significant (p=0.319). Mean EQ for 1st, 2nd and 3rd clinical years were 104.4±13, 104.4±20, and 107.1±12. This rise with increasing years was however not significant (p=0.659). EQ showed a weak positive correlation with age (r=0.1) but this was not significant (p=0.29). We report low EQ among students of the UGMS with no significant difference between gender, age and clinical year. EQ should be actively taught as part of the curriculum in UGMS. A larger study involving other medical schools in Ghana is recommended.

**Funding:**

None Declared

## Introduction

An emotion is a complex state that consists of a subjective experience, a physiological reaction and an expressive reaction. The third edition of the Oxford Advanced Dictionary defines intelligence as ‘the ability to learn, understand and think in a logical way about things’. Emotional intelligence is therefore the ability to comprehend one's own feelings, to listen and perceive the emotions of others and feel them, and to express this emotional state in a useful way.^1^ Emotional intelligence (EQ) is characterized by the ability to control urges, to limit impatience, to adjust one's mood to effectively prevent frustration, to curb the inability to think, to be able to empathize and hope.^2^ The new model defines EQ as “the ability to reason validly with emotions and with emotion-related information, and to use emotions to enhance thought”. These abilities involve identifying emotional content, facilitating thinking, understanding meanings of emotions, and managing emotions.^3^

Empathy on the other hand is another important part of the doctor-patient relationship and it is one of the elements of emotional intelligence that demands special attention. In the medical perspective, Mercer et al define it as “ability to: (a) understand the patient's situation, perspective and feelings (and their attached meanings); (b) to communicate that understanding and check its accuracy and (c) to act on that understanding with the patient in a helpful (therapeutic) way”.^4^

The importance of empathy cannot be overemphasized however it has been shown that some humanistic attributes such as empathy are influenced by one's demographic factors and it is known to decline during the medical school training more especially during the clinical clerkship program.^5^ Edward L. Thorndike, an American psychologist, proposed the concept of social intelligence in 1920.

He described his concept as the ability to understand men and women, managing them and acting wisely in human relations.^6^ His concept formed the foundation of emotional intelligence. In 1950, Abraham Maslow described the hierarchy of needs. At the top of this list, he placed emotional needs such as friendship, family, self-esteem and self-actualization above safety and physiological needs.^7^

The term ‘Emotional Intelligence’ was coined by Peter Salovey (a social psychologist) and John D. Mayer (a personality psychologist) in 1990 and they described the concept as a form of interpersonal intelligence that involves the ability to monitor one's own feelings and emotions as well as those of others, and differentiated from the various emotional states by the ability to use this information to direct one's thinking and action.^8^

David Gorman describes emotional intelligence as comprising of five components. The first component is Self-awareness (knowing yourself) and the hallmark of this element is self-confidence and realistic self-assessment. Recognition of one's own emotions is the primary step to understanding personal emotions. The second component is Self-regulation, that is, the ability to control our emotions. This is characterized by commitment, optimism and a strong desire to succeed. It allows us to overcome regrets and setbacks.

Motivation is the third component of emotional intelligence and it is described as the ability to control and direct our emotions to achieve an objective. This element is a step beyond self-regulation and hence highly motivated individuals tend to be more effective and productive.

Empathy, the fourth component, is the ability to recognize other people's emotions. Empathetic people are better at professions relating community outreach, teaching, sales and administration because they are able to pick up subtle signals from people which point to what others really need.^9^ The skill of empathy should therefore characterize every health professional for the best outcome in clinical practice.

The fifth and final component is Social skills. This is the ability to handle the emotions of others. Individuals who have harnessed this skill tend to be good leaders and are popular. Excellent social skills help in the establishment of rapport and the management of relationships. This is a quality that is welcomed in the medical profession.

The life of a medical student is very formidable, and it requires hard work and commitment. The medical student is expected to learn large volumes of information within a short period of training. In addition, the medical student is always subjected to a series of assessments and presentations as part of the training. The training is characterized by short hours of sleep, long hours of studying, and a huge pressure to succeed.^10^ It is therefore not surprising that stress and anxiety are constantly being experienced by medical students and this may affect their cognitive and learning functions.^11^

One way of coping with the pressure of medical school is by developing one's emotional intelligence. It has been shown that students with high emotional intelligence found examination periods less threatening.^12^ A study by Gangal et al reported that emotional intelligence correlates negatively with stress and it is useful in the management of stress at the work place.^13^ It has also been documented that students with a high emotional intelligence find presentations and public speaking less unnerving.^14^ Marquez et al also found a significant positive correlation between emotional intelligence and academic performance as well as pro-social behaviour.^15^ Additionally, it has been documented in Kentucky that medical students with high emotional intelligence are better at performing physical examination on patients due to their high empathic concerns.^16^

These studies have shown the potential benefits of emotional intelligence. The development of one's emotional intelligence may not only reduce the workload of the medical student, but it will definitely make the training program more bearable. It therefore goes without saying that efforts must be made to actively develop the emotional intelligence of medical students.

This is because emotional intelligence improves one's ability to establish a rapport with patients, make them feel comfortable and provide the much-required emotional support. Emotional intelligence can also be used to establish a trusting relationship and elicit satisfaction in the doctor-patient interaction.

A study was conducted by Smrithi et al (2013) in Bangalore where the emotional intelligence of 150 medical students was assessed using the self-assessment score.^17^ They found that 66% of the students had a good emotional intelligence. The study was also consistent with the findings of Imran et al (2013), where the average medical student had an above average emotional intelligence.^18^ There evidence that developing emotional intelligence in nurses may have a positive impact on certain caring behaviours.^19^ It has also been documented among nurses that high emotional intelligence corresponds with good leadership skills.^20^

This study was undertaken to explore the level of emotional intelligence among medical students of the premier medical school in Ghana. As far as the authors are aware, this assessment has not been done among medical students anywhere in Ghana. Furthermore, the tool for assessing emotional intelligence has not been tested in this population. This assessment will therefore form the foundation for evaluating the curriculum of our medical school to ensure that it addresses issues of emotional intelligence in a bid to better equip products of the school for the healthcare career ahead. It will also afford the students the opportunity to have an objective evaluation of their emotional intelligence status which could assist in addressing coping challenges they may be facing in the academic, family or personal lives.

## Methods

A cross-sectional study was conducted on the Korle Bu campus of the University of Ghana Medical School (UGMS) in the city of Accra. The UGMS is one of the six schools under the College of Health Sciences of the University of Ghana. The School runs two programmes - a traditional six-year course and a new four-year graduate entry medical programme (GEMP). The traditional programme attracts students qualifying from the senior high school system after 12 years of education whilst the GEMP program enrols students after their first degree in the sciences (16 years of education). Both programmes are divided into the pre-clinical and clinical programmes. The clinical programme is further divided into the first, second and third/final clinical years. The UGMS also runs a similar six-year and four-year programme for dentistry students. Medical and dentistry students in the clinical programme were targeted for this study. The current enrolment of the medical school stands at 583 for clinical year students as at the beginning of the 2014/2015 academic year. A total of 242 students were approached to enrol in this study. Only 111 students, randomly selected from each clinical year group, completed the questionnaire. Clinical year students who had repeated any year in the previous five years were excluded from the study.

The questionnaire consisted of 2 sections - Demography and the Emotional Quotient Self-Assessment Checklist (EQSAC).^21^ The first section consisted of the demographic details which included the age, gender and clinical year. The second section assessed the ESQAC designed by Sterrett. It consisted of 30 statements, five each for the areas for self-awareness, self-regulation, motivation, empathy, and social competency.

This is a five-point Likert rating scale, ranging from 1 to 5; total score on each of 6 facets range from 5 to 25 and a cut-off value for good emotional intelligence is 20 in each domain, the score below which needs improvement in the respective domain. Total score of EQ including all the domains ranges from 30 to 150.

For differences in the distributions of proportions, Chisquare tests were carried out. For the mean differences, ttests and Analysis of Variance (ANOVA) were used where assumptions underlying them were met, otherwise their non-parametric equivalents of Wilcoxon Rank Sum and Kruskal-Wallis tests respectively were used. Cutoff points for significance levels were 5% (p<0.05), 1% (p<0.01) and 0.1% (p<0.001). For findings that were not significant, exact p-values are quoted.

## Results

**Figure 1:**
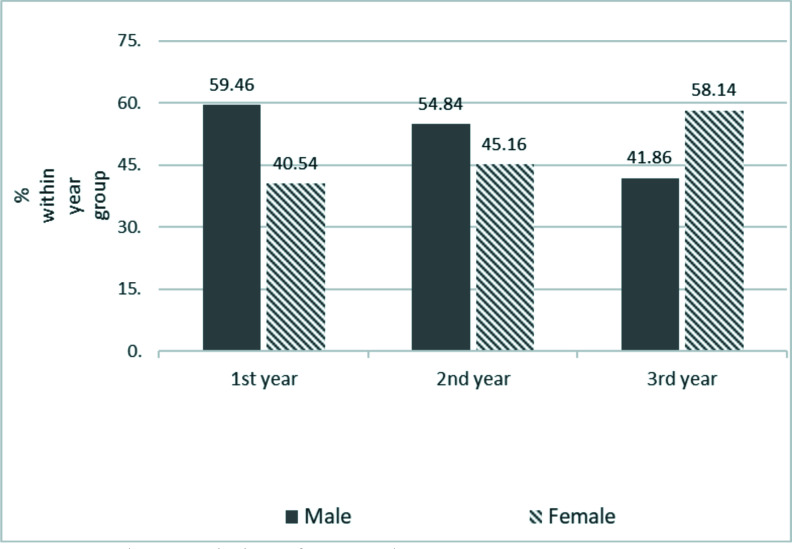
Characteristics of respondents

A total of 242 medical students were recruited however 111 completed and returned the questionnaire giving a response rate of 45.9%. Average age was 24 years ±1.5 years. Overall, there were more males than females and the largest group of respondents came from those in the 3rd clinical year. (Table 1) The gender distribution across the year groups is also shown below. There were more male than female respondents in all year groups except 3rd year. (Figure 1)

**Table 1:** Background characteristics of respondents

Clinical Year	Males	Females	N (%)	Mean Age (SD)
**1st Clinical**	22	15	37 (33.3)	23.38 (1.62)
**2nd Clinical**	17	14	31 (27.9)	23.81 (1.25)
**3rd Clinical**	18	25	43 (38.8)	24.81 (1.35)
**Total**	57	54	111 (100)	24.05 (1.54)

For global score, 16 students out of 111 (14.1%) had good emotional intelligence (>120) with a range of scores from 120–128. Mean total empathy score for all 111 participants was 105.49 (SD=14.84)

**Figure 2:**
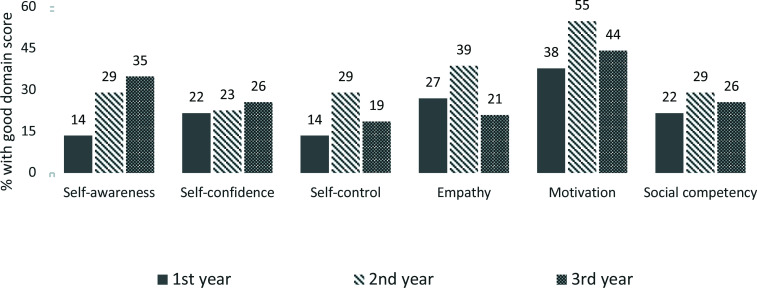
Domain Scores for Respondents with Good Emotional Intelligence by clinical years

Overall, the EQ domain with the best performance was motivation with self-control having the least as shown in the above table. (Table 2) The proportions with good EQ score within each year group is shown below. (Figure 2) For example, 14% of first year students had good selfawareness compared to 29% of second year students and 35% of third year students.

**Table 2:** Proportion of medical students with good emotional intelligence

	N (%)	
EQ Domain	Not good	Good
**Self-awareness**	82 (73.9)	29 (26.1)
**Self-confidence**	85 (76.6)	26 (23.4)
**Self-control**	89 (80.2)	22 (19.8)
**Empathy**	80 (72.1)	31 (27.9)
**Motivation**	61 (54.9)	50 (45.1)
**Social competency**	83 (74.8)	28 (25.2)

In terms of proportions, gender only played a significant role on self-awareness (p<0.05) compared to the other EQ domains with females scoring better (35.2% good) compared to their male counterparts (only 17.5% good) within that domain based on the cut-off point of 20/25 as stated in the literature. (Table 3) This means that 64.8% of females and 82.5% of males performed poorly in self-awareness. The same logic applies to the other domains whose differences were not statistically significant. For mean scores of EQ, the domain of Motivation recorded the highest scores for both male and female with the females having a slightly higher score. (Table 4) Females had higher scores in all domains except Social competency, where the males dominated.

**Table 3:** Influence of gender on emotional intelligence based on proportions

	EQ - Number (%)			
EQ Domain	Male N=57	Female N=54	Total N=111	p-value
**Self-awareness**	10 (17.5)	19 (35.2)	29 (26.1)	<0.05*
**Self-confidence**	15 (26.3)	11 (20.4)	26 (23.4)	0.460
**Self-control**	10 (17.5)	12 (22.2)	22 (19.8)	0.537
**Empathy**	16 (28.1)	15 (27.8)	31 (27.9)	0.973
**Motivation**	26 (45.6)	24 (44.4)	50 (45.1)	0.901
**Social competency**	16 (28.1)	12 (22.2)	28 (25.2)	0.478

**Table 4:** Influence of gender on emotional intelligence based on means

	Mean (SD) of EQ			
EQ Domain	Male	Female	Total	p-value
**Self-awareness**	17.2 (3.2)	17.9 (3.3)	17.5 (3.3)	0.489
**Self-confidence**	17.3 (3.1)	17.4 (2.6)	17.4 (2.9)	0.695
**Self-control**	17.0 (3.2)	17.3 (2.6)	17.1 (2.9)	0.801
**Empathy**	17.3 (3.2)	17.9 (2.6)	17.6 (3.0)	0.289
**Motivation**	18.6 (3.5)	18.8 (2.4)	18.7 (3.0)	0.936
**Social competency**	17.4 (3.4)	16.9 (3.1)	17.2 (3.3)	0.234
	Mean (SD) for Males	Mean (SD) for Females		p-value
**Summary**	104.82 (17.07)	106.19 (12.20)		0.319

There were no statistically significant differences between gender in the mean EQ score for all the domains as seen from the p-values. (Table 5)

**Table 5:** Influence of the level of clinical experience on emotional intelligence: Based on proportions

	N (%) with good EQ				
EQ Domain	1^st^ year (N=37)	2^nd^ year (N=31)	3^rd^ year (N=43)	Total (N=111)	p-value
**Self-awareness**	5 (13.5)	9 (29.0)	15 (34.9)	29 (26.1)	0.087
**Self-confidence**	8 (21.6)	7 (22.6)	11 (25.6)	26 (23.4)	0.909
**Self-control**	5 (13.5)	9 (29.0)	8 (18.6)	22 (19.8)	0.270
**Empathy**	10 (27.0)	12 (38.7)	9 (20.9)	31 (27.9)	0.240
**Motivation**	14 (37.8)	17 (54.8)	19 (44.2)	50 (45.1)	0.370
**Social competency**	8 (21.6)	9 (29.0)	11 (25.6)	28 (25.2)	0.780

There were no significant differences in the proportions that had good EQ domain scores between the different clinical year groups as seen from the p-values above, even though the 2nd year clinical students seemed to have the best performance overall. (Table 6) They had lower scores than the third-year group in the self-awareness domain however their scores were consistently higher than those for the first-year group in all domains.

**Table 6:** Influence of the level of clinical experience on emotional intelligence: Based on means

	Mean (SD) of EQ				p-value
EQ Domain	1^st^ year	2^nd^ year	3^rd^ year	Total	
**Self-awareness**	17.1 (2.5)	17.4 (4.0)	18.0 (3.2)	17.5 (3.3)	0.298
**Self-confidence**	17.3 (2.5)	16.7 (3.7)	17.9 (2.4)	17.4 (2.9)	0.490
**Self-control**	17.3 (2.3)	16.8 (4.1)	17.2 (2.4)	17.1 (2.9)	0.957
**Empathy**	17.5 (2.6)	17.9 (3.7)	17.4 (2.7)	17.6 (3.0)	0.381
**Motivation**	18.2 (3.4)	18.8 (3.7)	19.0 (1.9)	18.7 (3.0)	0.394
**Social competency**	16.9 (3.1)	17.0 (3.9)	17.5 (3.1)	17.2 (3.3)	0.656

No significant differences in the means of respondents that had good EQ domain scores between the different clinical year groups. The 3rd year clinical students seemed to have the best performance overall. They had lower scores than the second-year group in the Empathy domain however their scores were lower than the first-year group in two domains - Self-confidence and Self-control. There was a steady rise in emotional intelligence with increasing experience in the clinical years however this difference was not statistically significant.

## Discussion

The response rate for this study was 49.6%. This is comparable with a study done by McKinley in 2014 in the USA who recorded a response rate of 42.8% but much higher than Todres et al who recorded a response rate of 12.3%. The generally low response rate compared with the globally accepted standard of 70% is quite prevalent because assessment of emotional intelligence requires completion of very long questionnaires.^22^

### The Emotional Intelligence of Medical Students

The mean age and mean emotional intelligence score of the average medical student of the University of Ghana Medical School is represented in Tables 1 and 2. The study revealed that the majority of medical students had a low emotional intelligence score which consistent with Imran et al but contrary to another study by Smrithi et al.^17^,^18^ It is worrisome that a majority of our future doctors have low emotional intelligence. From the study, one can infer that the average student has poor coping mechanisms to deal with the enduring stress associated with the medical training program which will ultimately spill into the medical profession when they graduate.^10^

Emotional intelligence enables one to harness his or her emotions to improve cognition. Based on this study, it can be inferred that the average medical student cannot competently handle his or her emotions during challenging moments such as examination which may lead to poor academic performance.^16^ It is known that emotional intelligence is an important ingredient in the delivery of high quality patient-centred care due to its influence on factors such as the establishment of a rapport, taking a clinical history, making an accurate diagnosis, treatment and advice on lifestyle changes.

This is due to the fact that it identifies and appeals to the emotional state of the patients.^23^,^24^ From this, it is clear that if interventions are not put in place, the capacity of the medical student to deliver the new standards of high quality patient care will at best be pale. Patient satisfaction will be poor which will open doors of malpractice lawsuits.^25^–^27^

### Emotional Intelligence and Gender

There have been conflicting research findings regarding emotional intelligence and gender. Todres et al^28^ and Smrithi et al^17^ both reported higher emotional intelligence scores in females compared with males. Carr^29^ and Ajmal et al^30^ on the other hand, reported the reverse, that is, emotional intelligence is higher in males. Furthermore, a thesis dissertation by McKinley, who used resident medical doctors in Harvard, reports no difference in global emotional intelligence between males and females.

Her findings corroborate with earlier study conducted by Salovey et al^31^ and Bar-On in 2000.^32^ This study found no significant difference in the emotional intelligence of the male and female medical students (Table 3 and 4).

Upon further examination of the effect of gender on emotional intelligence, this study reports that there was no significant difference between males and females within each of the five domains of emotional intelligence. Results obtained from Fernandez-Berrocal et al study showed that emotional intelligence is higher in women than men with age being the main mediator without which there is no significant difference between the two sexes.^33^ In the absence of other demographic variables, gender has an influence on the emotional intelligence of an individual.^33^,^34^

In view of this, the difference between males and females was further explored in this study. The mean age of the students is as described in Table 1. There were no significant differences between the males and females in terms of age (Table 2), thus the insignificant difference in emotional intelligence between the male and female medical students is a true representation of the influence of gender on EQ. The overall average mean score for emotional intelligence was however higher in females though not statistically significant. This correlates well with several studies^17^,^28^,^35^ and contradicts several other studies too.^22^,^29^,^30^,^36^,^37^

### Emotional Intelligence and Clinical Year

This study showed an increase in the mean emotional intelligence score with regard to the clinical year of the student (Table 6). The final clinical year students had the highest mean emotional intelligence score followed by the 2nd clinical year students with the 1st clinical year students scoring the least. However, these differences were not significant (Table 5). It was also shown that there was no significant difference in the emotional intelligence between the first clinical years and the final clinical year medical students. (Table 6) These results were not similar with the findings of a study conducted in the Putra Medical School in Malaysia, where it was found that final year medical students had a significantly higher emotional intelligence than the first year medical students.^38^ The current study findings are however, consistent with that of Imran et al in Pakistan, where there was no significant difference in emotional intelligence between the final clinical year and the 1st clinical year students.^18^ A plausible explanation for these results according to Imran was the fact that the medical curriculum focuses more on the development of clinical skills as against the acquisition of interpersonal skills. According to Chew et al, a high emotional intelligence among the final year medical students is a strong indication of the toughness and humanism developed during the medical school training program.^38^

In this study, the insignificant difference in the emotional intelligence with respect to the level of clinical experience is reflection of the poor impact of the medical curriculum in actively stimulating the emotional awareness of the students. One could say that there is an element of passiveness in the development of the emotional intelligence of the students. In other words, the students pick up little elements from their experiences in the school that may increase their emotional intelligence. This passive approach is not enough to significantly build their emotional intelligence during their training. From this study, it can be implied that doctors are churned out every year with virtually the same level of emotional intelligence which they came into the School with.

### Emotional Intelligence and Age

This study found an insignificant and weak positive correlation of 0.10 (p=0.29) between emotional intelligence and age. This result contrasts with findings in literature where the older medical students had significantly higher scores.^28^,^39^ The results of this study did not also tally with the findings of Fariselli et al who reported a significant but a weak positive relationship between the age of individuals in the population and their respective emotional intelligence.^40^ For a sample taken from the general population with a wide age spectrum, there are stronger factors besides age that affect the emotional intelligence of individual. With this thought in mind, it can be said that the comparatively narrow age range of medical students may be so limited that the positive influence of age on emotional intelligence could not be attained.^33^,^40^

## Conclusion

This study reports low emotional intelligence scores among students of the UGMS with no statistically significant difference between gender, age and clinical year. The authors recommend that emotional intelligence should be actively included in the curriculum of the UGMS. A larger study involving other medical schools in the country will also be useful.

## Figures and Tables

**Table 7:** ANOVA analysis of clinical year and mean EQ scores

Clinical Year	Mean Score	Std. Dev	F (ANOVA)	Sig
**1^st^ Clinical**	104.40	13.19	0.419	0.659
**2^nd^ Clinical**	104.51	20.09		
**3^rd^ Clinical**	107.11	11.63		
